# Hospital Dispute at Torquay

**Published:** 1902-04-12

**Authors:** 


### HOSPITAL DISPUTE AT TORQUAY.

An unfortunate dispute has arisen at Torquay in connection with the Torbay Hospital. Last year at the annual meeting, by 20 votes to 9, the subscribers passed a new rule: "That the honorary staff shall consist of three physicians, three surgeons, an ophthalmic surgeon, an anæsthetist, and a dental surgeon. They shall be elected annually, as vacancies occur, by a special board, and shall be eligible for re-election." The medical men at the time strongly objected to the proposal that their appointments should be held from year to year, and the climax has now come through the action of the "special board," who recently re-elected the whole of the honorary medical staff, with two exceptions—these being gentlemen who had honourably served the hospital for many years. The doctors of the Torquay Medical Society—25 in number—subsequently met and pledged themselves not to apply for the vacant posts. On Friday, as the outcome of this, there was a representative meeting of governors of the hospital to consider the question of repealing the offending rule and substituting for it one which provided for the filling of vacancies as they occur. Mr. W. H. Kitson presided over the gathering, and the proceedings were characterised by a good deal of angry discussion. The chairman argued that the action of the medical men was practically one of ignoring the calls of humanity for mere professional status, and an attempt to pass a vote of censure on the Board of Management. Capt. Phillpotts, the former M.P. for the Division, characterised the existing rule as improper, unfair, and derogatory to the medical profession, and Dr. Gardner demanded that it should be rescinded before the medical men would negotiate with the Managing Board. Dr. Pollard declared that an abominable injustice had been perpetrated, and added that he had had a good many years' experience of hospitals, but had never yet found a hospital that treated its honorary medical officers with less courtesy. Dr. Stebb entered a protest against the secret ballot in the election of the medical staff, and hinted that if the weekly Board resigned there would be found able administrators, competent people, to conduct the affairs of the hospital long after the fame of those who were present was simply preserved by stained-glass windows in all the churches. Some more warm discussion followed, and after a debate of two and a-half hours, it was agreed to adjourn for a month, in order that a conference might ensue between the Board and members of the Torquay Medical Society.

